# Exploring Bioinspired Climatic Design Strategies for a Low-Carbon Future: A Case Study of a Hot–Humid Climate in Sri Lanka

**DOI:** 10.3390/biomimetics10100671

**Published:** 2025-10-06

**Authors:** Arosha Gamage, Anir Upadhyay, Richard Hyde

**Affiliations:** 1 Faculty of Architecture, Design & Planning, University of Sydney, Sydney, NSW 2006, Australia; richard.hyde85@gmail.com; 2 School of Built Environment, University of New South Wales, Sydney, NSW 2052, Australia; anir.upadhyay@unsw.edu.au; 3 Department of Arts, Design and Architecture, UNSW College, Sydney, NSW 2052, Australia

**Keywords:** bioclimatic design, bio-inspired design, Bioinspired Climatic Design (BCD), climate analysis, biomimicry, biomimetic, biophilia, hot–humid climate, low carbon design, ecological sustainability

## Abstract

Bioclimatic design, rooted in vernacular architecture, aims to create buildings that harmonise with their local climate and context. Over the past five decades, continuous advancements have strengthened its foundation for climate-responsive architecture. However, the development of bioinspired thinking extends new opportunities to enhance ecological sustainability and innovation in bioclimatic design. This study introduces Bioinspired Climatic Design (BCD) as an advancement of bioclimatic design, integrating ecological processes, human behaviour, and high-resolution climate data to create sustainable, climate-responsive low-carbon architecture. Focusing on residential buildings in hot–humid climates, it categorises BCD strategies into primary and modifying adaptive approaches, examined through four case studies using observation and spatial analysis. Findings emphasise the importance of aligning design with climate, ecology, and occupant behaviour to achieve low-carbon, resilient architecture, especially in challenging conditions. The research calls for a paradigm shift from conventional climate-responsive design towards a holistic, ecologically integrated framework for future-oriented built environments.

## 1. Introduction

Vernacular architecture demonstrates a nuanced response to climatic and microclimatic conditions through the deployment of locally available materials, indigenous construction technologies, and the expertise of local craftsmen [1,2,3]. Examination of its historical evolution reveals underlying scientific principles that continue to inform innovative approaches to building morphology, typologies, elements, and technologies, thereby offering valuable strategies for bioclimatic design and the advancement of low-carbon architecture [4,5]. However, in contemporary practice, the direct replication of traditional forms and materials is neither feasible nor practical, given the transformation of building functions, shifts in typological frameworks, evolving user expectations, material scarcity, diminishing artisanal knowledge, the prevalence of industrialised alternatives, accelerated patterns of urban development, and increasingly stringent regulatory frameworks [6].

Consequently, architectural design variables must be critically reinterpreted and systematically organised along a climatic continuum, enabling contextually responsive solutions that account for variation in form, geometry, materiality, and architectural expression across diverse environmental settings [4,7].

The onset of the Industrial Revolution marked a decisive break from these climate-responsive traditions. The growing demand for large-scale industrial and residential buildings encouraged the integration of technology and modern materials. This shift culminated in the International Style, which was enabled by fossil fuel energy and mechanical systems, and promoted universal prototypes of concrete and glass that largely disregarded local climatic conditions. Consequently, buildings were conceived as climate modifiers rather than climate-responsive forms [8].

While this movement thrived on fossil fuels, a minority of architects, including Frank Lloyd Wright, Maxwell Fry, Jane Drew, Charles Greene, and Henry Greene, advocated for climate-responsive design. Fry and Drew’s Tropical Architecture [9] remains a landmark illustrated passive principle tailored to warm-humid climates, laying foundations for modern bioclimatic design. While technology expanded architectural possibilities, it often did so at the expense of ecological sensitivity.

Olgyay also proposed an analogy between plant morphology across different climatic zones and the optimal form and shaping of buildings [10], an idea that later informed the concept of Bioinspired Climatic Design (BCD). Despite its potential, limited research has examined the integration and interrelationships of these concepts within architectural discourse [11,12,13]. Bioinspired design more broadly investigates analogies between living organisms and their biomes to enhance both human and ecological well-being. This involves understanding the complementary relationship between biomimicry and biophilia in architectural practice [14,15,16]. Biomimicry emphasises the emulation of nature’s forms, processes, and ecosystems to generate design strategies [17,18,19], while biophilia focuses on fostering human connections with nature to promote psychological comfort and well-being [20,21]. BCD synthesises these approaches by examining how organisms adapt to their local environments and applying such strategies to architectural design. In practice, this translates into solutions for temperature regulation, air movement, lighting, energy efficiency, and waste reduction, thereby advancing outcomes that benefit both human and ecological systems [19,22]. The research aims to bridge the gap between theoretical principles and real-world practice by equipping designers with a framework to interpret climatic data together with nature-based solutions as an advancement to bioclimatic design. Unlike generic sustainability guidelines, which often remain abstract, this approach emphasises contextual adaptability and ecological integration.

## 2. Background

### 2.1. Vernacular Architecture and Bioclimatic Design

Vernacular architecture across the world illustrates how built forms evolved as direct responses to local climate, available resources, and cultural practices [5,23]. These traditional solutions were not arbitrary; rather, they represented adaptive strategies refined over generations. Rudofsky’s Architecture Without Architects [24] highlighted the ingenuity of early construction methods, which employed techniques such as maximising solar gain in winter, providing shading in summer, and enabling natural ventilation to maintain thermal comfort and indoor air quality. Importantly, these principles remain highly relevant today as the building sector seeks low-carbon solutions to address climate change.

### 2.2. Bioclimatic Approach and Design Strategies for a Hot–Humid Climate Towards a Low Carbon Future

Bioclimatic design engages contextual architectural strategies that respond to local climatic conditions to optimise comfort and energy efficiency. Core climatic variables include temperature, humidity, wind, solar radiation, and rainfall, while microclimatic factors such as site-specific geography and localised environmental conditions further influence design outcomes. Accounting for seasonal and diurnal variations enables the development of context-specific strategies for achieving thermal comfort. Analytical tools, including bioclimatic charts and computer simulations, provide critical frameworks for formulating such a strategy. Foundational contributions by Olgyay [10], Givoni [25,26], and Szokolay [27] established the use of psychrometric charts as a basis for climate-responsive design. Nonetheless, contemporary practice often continues to rely on experiential knowledge, heuristic methods, building codes, and culturally embedded norms in design decision-making.

Hot–humid climates, defined by high temperatures, humidity, and minimal diurnal variation, present challenges for thermal comfort [4]. While occupants of naturally ventilated buildings often adapt to higher heat and humidity [28,29,30,31], passive strategies alone may be inadequate where airflow is restricted, particularly in dense urban settings. Recommended approaches include elongated north–south building forms, lightweight shaded envelopes, and cross-ventilation [4,10,26,32,33], complemented by ventilative and evaporative cooling, earth cooling [4], well-proportioned courtyards, thermal mass with night flushing [29,34], shading devices [35], and urban-scale strategies such as optimised street canyons, “shadow umbrellas”, and the Local Climate Zone (LCZ) framework for climate-sensitive planning [34,36,37].

### 2.3. Towards Bioinspired Climatic Design (BCD)

Buildings account for 30–40% of global energy use and 26% of carbon emissions, making the sector central to climate action [38]. While bioclimatic design reduces operational energy through passive strategies and local materials, bioinspired design advances this vision by integrating biomimicry and biophilia.

Building upon the principles of bioclimatic design, Bioinspired Climatic Design (BCD) represents a further evolution in climate-responsive architecture. Unlike earlier approaches, BCD integrates biomimicry and biophilia to embed ecological intelligence into the built environment. Biomimicry draws on nature’s time-tested strategies for environmental performance [17,39], while biophilia incorporates natural patterns, elements, and systems to strengthen human connections with nature, thereby enhancing psychological and physiological well-being [20,21]. When combined with bioclimatic principles, these strategies aim to create energy-efficient, low-carbon, and contextually responsive architecture that fosters deeper ecological integration [13,39]. Drawing on nature’s adaptive strategies, BCD promotes energy efficiency, reduces embodied carbon, and fosters resilience by harmonising architecture with ecosystems. Beyond immediate energy savings, BCD offers a transformative pathway toward a low-carbon future, addressing climate change and biodiversity loss while enhancing human well-being through ecological integration [10,13,39].

**Figure 1:**
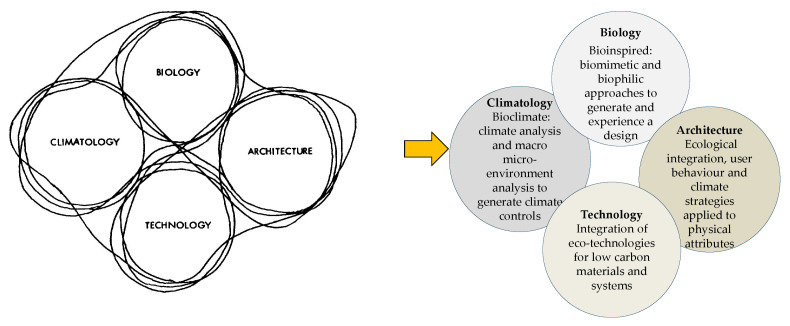
Olgyay’s model [10], interlocking fields to Gamage’s model’s [22] expanded fields.

The intellectual roots of this approach can be traced to Victor and Aladar Olgyay’s pioneering bioclimatic methodology in the early 1950s, which positioned architecture at the intersection of climate, biology, and technology [10] (Figure 1). Although their model hinted at these interconnections, it lacked a comprehensive framework for ecological integration. Contemporary adaptations address this gap by emphasising biomimetic concepts, biophilic patterns, and eco-technical innovation [22].

**Figure 2:**
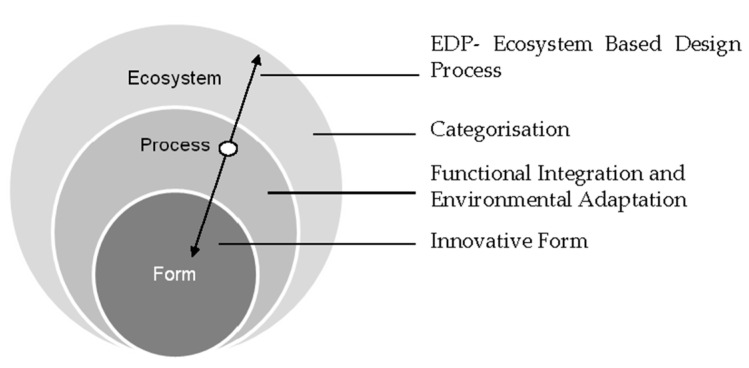
Biomimicry theoretical model [40].

While Olgyay proposed an analogy between plant morphology across climatic zones and the optimal shaping of buildings [10], systematic research on the integration and interrelationships underpinning BCD remains limited. To address this limitation, the Biomimicry Theoretical Model [40] (Figure 2) introduces a multidimensional framework (Table A1 in Appendix A) that operates across multiple scales, i.e., from ecosystem-level systems to process-based functions and form-making and incorporates both direct and indirect design strategies.

Indirect approaches use biophilic design elements [41,42], natural climatic variables (air, rain, light, wind, temperature, humidity), and ecosystem-level biomimicry [19,43] to inform building layout, spatial configuration, envelope design, building form, structure, and material selection. Collectively, these strategies promote resilience, human comfort, and ecological sustainability. This study extends their framework by incorporating ecological integration, biomimetic strategies, biophilic design patterns, and eco-technological solutions [22].

### 2.4. Research Gap and Limitations

The existing literature on bioclimatic design often assumes ideal site conditions, neglecting the complexities of dense urban fabrics and varied topographies. This highlights the need for adaptable, site-specific strategies that integrate biomimetic, biophilic, and bioclimatic principles. Current recommendations for hot–humid climates are largely generic; less attention has been given to context-sensitive approaches that reconcile ecological integration with real-world constraints. This study addresses the gap through four residential projects in Colombo, Sri Lanka, evaluating Bioinspired Climatic Design (BCD) strategies that respond to both climatic and user-specific needs.

The study is exploratory and limited by the absence of quantitative validation through climate simulations, energy data, or user surveys. Findings are based on designers’ reasoning, observation of case study buildings, and spatial analysis using drawings and photos. The study does not measure the performance equivalence between natural features and architectural forms. However, biological analogies, such as mimicking the large, funnel-shaped leaves of tropical plants through deep overhangs for shading and rainwater drainage, are considered at a conceptual rather than quantifiable level.

### 2.5. Research Aim and Approach

This study aims to identify appropriate BCD strategies that are not only climate-appropriate but also adaptable to site-specific and user-specific challenging conditions. By doing so, the study intends to contribute to the development of applied design strategies for hot–humid climates, advancing the discourse from generic guidelines toward context-sensitive, low-carbon architecture.

This paper adopts a mixed-methods approach to explore BCD strategies in hot–humid climates, focusing on case studies in Colombo, Sri Lanka. The methodology integrates three key components:

- Climate Analysis—Examining macroclimatic and microclimatic conditions and their influence on architectural form.
- Case Study Investigation—Analysing selected residential projects that incorporate vernacular, bioclimatic, and bioinspired (biomimicry and biophilic) principles to evaluate performance and design outcomes.
- Design Application—Proposing site-specific BCD strategies informed by both climate data and ecological analogies, with emphasis on practical applicability in contemporary contexts.

## 3. Research Method

**Figure 3:**
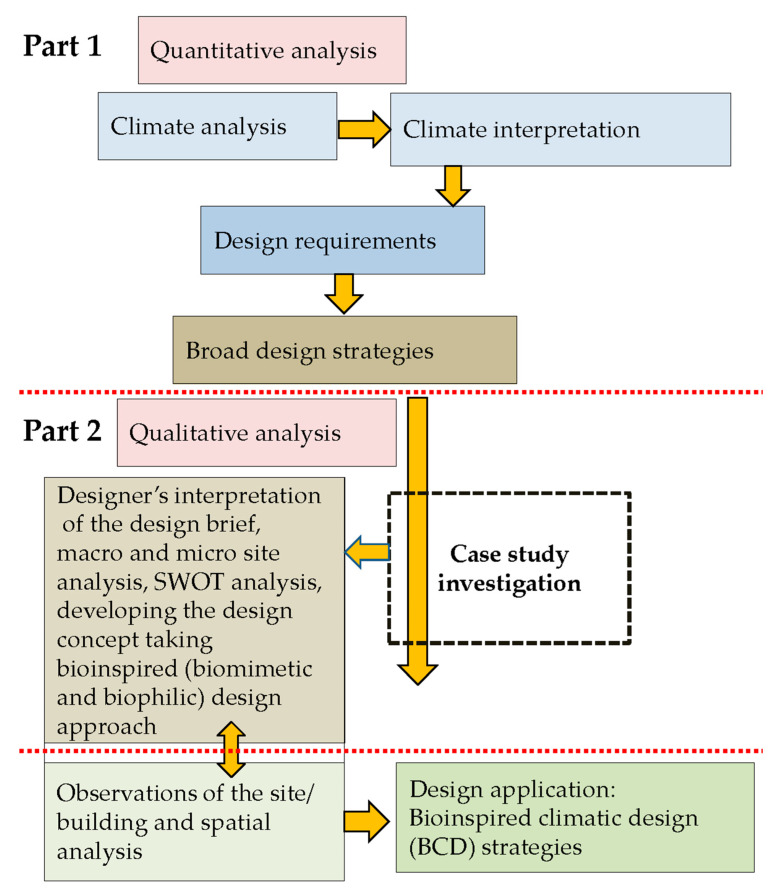
Research methodology.

This research focuses on Colombo, Sri Lanka, which is characterised by a warm, humid, tropical rainforest climate (Af) under the Köppen–Geiger classification [44]. To develop context-specific BCD strategies, a mixed-methods approach is employed that integrates quantitative climate analysis with qualitative case study investigation. High-resolution hourly climate data are utilised to capture diurnal and seasonal variations, including extreme conditions, while selected architectural case studies provide insights into design practices and applications that inform the development of BCD strategies (Figure 3).

### 3.1. Part 1: Quantitative Climate Analysis

The first part analyses Colombo’s climate using a Typical Meteorological Year (TMY) dataset from Ratmalana, representing the city’s current climatic conditions [45]. TMY datasets, widely used in building simulation and energy modelling, provide hourly records of climatic elements which serve as the basis for developing broad climate-responsive strategies tailored to Colombo’s current climate.

### 3.2. Part 2: Qualitative Case Study Investigation and BCD Evaluation

The second part explores how climate-responsive design strategies are applied in practice. Four residential projects located in Colombo and its suburbs were selected as case studies based on their exemplary design features and availability to access the site. Data was collected through designers’ interpretations of the design approach, architectural drawings, site visits, and direct observation of spatial and material configurations. This phase examines how site-specific climatic and geomorphological conditions shape design decisions, including ventilation, shading, and envelope strategies. Finally, it evaluates the integration of bioinspired design principles of biomimicry and biophilia [18,40] within the case studies. Particular attention is given to how designers translate ecological analogies into architectural features, such as passive cooling, water management, shading, and indoor environmental quality. Material choices, spatial arrangements, and passive systems are analysed to determine their contribution to environmental performance and occupant well-being.

Together, these two parts provide a comprehensive methodology for developing applied BCD strategies in hot–humid climates. By combining empirical climate data with design practice and bioinspired thinking, the study bridges theoretical frameworks and practical solutions, offering transferable lessons for sustainable architecture in similar contexts.

## 4. Climate Analysis and Interpretations

Colombo’s Typical Meteorological Year (TMY) climate data were retrieved from a web-based repository, Climate.OneBuilding.Org. This climate file is created using 15 years of historical hourly weather data from 2009 to 2023. The TMY climate data set includes the hourly temperature, humidity, atmospheric pressure, sky condition, solar radiation, and wind speed and direction. While rainfall data is not included in the TMY data set, it remains essential for understanding seasonal variations in Colombo, as it can be more effectively analysed through wind and rainfall patterns. An hourly rainfall and wind data set was retrieved from Meteonorm software (Version 7) [46].

### 4.1. Understanding the Colombo Climate

**Figure 4:**
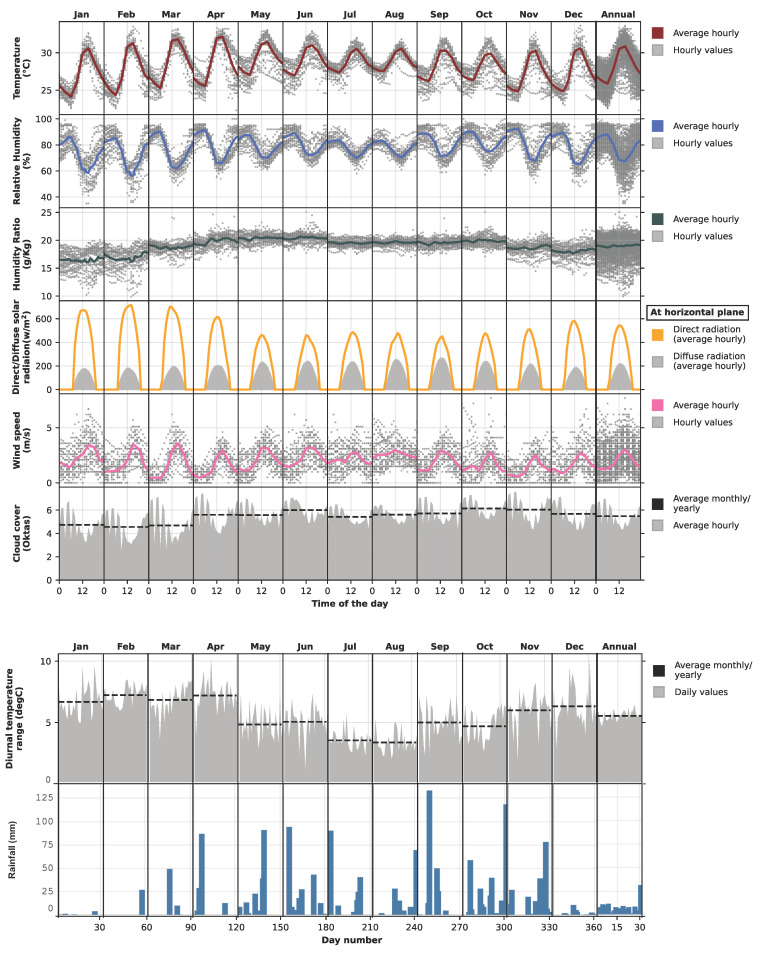
Climate graph for Ratmalana (Colombo).

From the TMY data set, the temperature in Colombo hovers between 24 °C and 32 °C, with a short period of temperature spikes, but not exceeding 35 °C (Figure 4). The highest diurnal temperature range is experienced from January to April (i.e., an average of 7degC), followed by November and December. In August, Colombo’s average morning temperature hovers around 27 °C, and the afternoon temperature reaches 30 °C. Average relative humidity varies between 70% and slightly over 80%. The highest temperature fluctuation in Colombo can be observed from November to February, and the lowest from May to August. The humidity ratio is useful for understanding the actual amount of moisture in the air. Colombo is predominantly humid, with average moisture content in the air exceeding 15 g/kg. For six months (May to October), the air moisture level remains significantly higher (>20 g/kg) than in the remaining months.

A significant variation in solar radiation is observed in Colombo. Months that receive less rainfall (December to April) demonstrate clear sky conditions with higher direct solar radiation, whereas rainy months (May to November) receive less direct solar radiation and more diffused solar radiation. The morning wind speed is relatively weaker than in the afternoon, which rarely exceeds 5 m/s. From May to August, the wind speed increases at night. The Colombo sky remains predominantly cloudy, particularly at nighttime. Colombo receives regular and sometimes intense rainfall for around seven months (May to November), while December to March receives the lowest rainfall.

**Figure 5:**
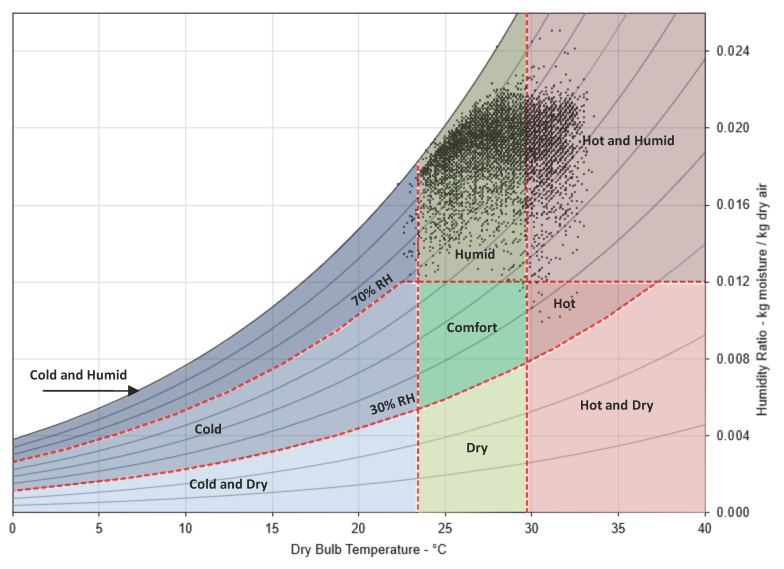
Psychrometric chart with hourly temperature and humidity data of Colombo and overlaid with various thermal environmental conditions.

Psychrometric charts have been widely used to show thermal environmental and comfort conditions. Hourly temperature and humidity data of Colombo have been plotted in the psychrometric chart (Figure 5), which has overlays of various thermal environmental conditions based on temperature and humidity [47]. The chart shows that Colombo experiences humid conditions for most of the year, followed by hot–humid conditions. This information is useful to understand the overall yearly environmental outlook. Based on the environmental conditions, as presented in the psychrometric chart, it can be concluded that there is little seasonal variation in temperature and humidity in Colombo.

However, the seasonal variation in Sri Lanka is defined by the monsoonal pattern. The Department of Meteorology, Sri Lanka [48] divides the weather in Sri Lanka into four seasons:

- First inter-monsoon season (March–April),
- Southwest monsoon season (May–September),
- Second inter-monsoon season (October–November) and
- Northeast monsoon season (December–February)

**Figure 6:**
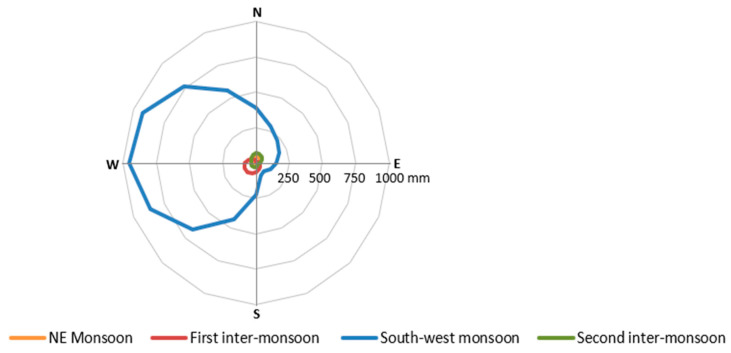
Wind-driven rainfall in four different seasons in Colombo.

The seasons are based on wind and rainfall patterns corresponding to each monsoon period. As its name suggests, the Southwest and Northeast monsoon periods experience dominant winds from the corresponding orientations. In contrast, Colombo experiences dispersed wind coming from all possible directions during inter-monsoon periods (Figure 6).

**Figure 7:**
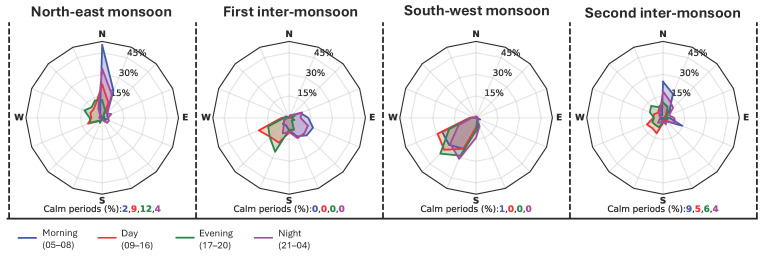
Wind roses: wind speed and directions in four seasons at different times of the day in Colombo.

However, based on wind direction and rainfall patterns, it is possible to distinguish two major seasons: wet (two inter-monsoon periods and the Southwest monsoon) and dry (the Northeast monsoon). Most importantly, the Southwest monsoon is dominant in Colombo as it brings a significant amount of rainfall, particularly wind-driven rainfall from the northwest to the southwest (Figure 7).

### 4.2. Understanding Seasonal Variations Using Temperature, Humidity, Cloud Cover, and Rainfall

**Figure 8:**
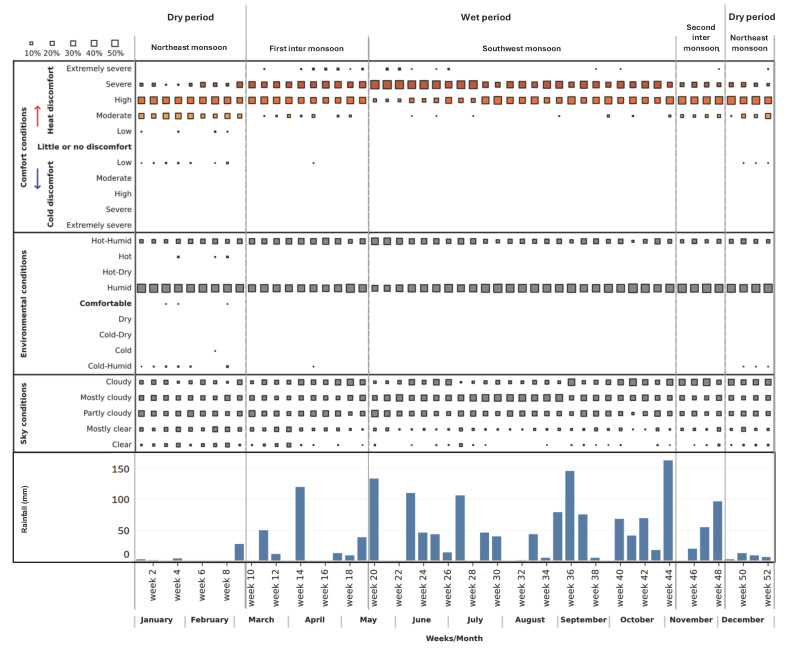
Weekly thermal environmental and comfort conditions, sky conditions, and rainfall patterns in Colombo. This helps to understand seasonal variations in Colombo.

To better understand the seasonal variations and the relationships between various climate elements, a weekly scale is used to plot thermal environmental conditions, comfort conditions, sky conditions and rainfall patterns (Figure 8). In Colombo, three months (December to February) are considered dry, as rainfall is scarce during those months. The dry period is also primarily humid with a high diurnal temperature range, moderate heat discomfort during the mornings, and a high level of discomfort during the daytime. The sky often remains clearer.

The wet period starts in early March and ends in November. At the beginning of the first inter-monsoon period, rainfall is infrequent but can get intense occasionally. Outdoor conditions are primarily humid, but frequent hot–humid conditions are also common during this period. A high level of discomfort may be experienced in the mornings due to heat, and this worsens (turning to severe discomfort) in the afternoons. The sky remains primarily cloudy in this period.

From mid-May until October, rainfall is significant due to southwest monsoon activity. During this period, southwesterly wind-driven rainfall is more common. Thermal environmental conditions remain humid, but the resultant comfort experience ranges from high to severe discomfort. The sky remains predominantly cloudy in this period. 

The second inter-monsoon period is a short transitional period between the wet and dry periods. The intensity of rainfall is often reduced during this period. Environmental conditions are humid, and as a result, people may experience high to severe heat discomfort most of the time. The sky remains cloudy during this period.

### 4.3. Bioclimatic Design Recommendations for Colombo

Colombo’s climate imposes a great challenge to the designers, as buildings always need to be protected from harsh environmental conditions. The sky remains mostly cloudy, with frequent rainfall making the situation worse by increasing relative humidity to a very high level. The humid outdoor environment, accompanied by low wind speed, can be detrimental to thermal comfort. Moreover, night-time and morning periods are significantly calm throughout the year, with wind speeds hovering around only 2 m/s. Due to a low diurnal temperature range, buildings with a high thermal mass may experience more discomfort than lightweight buildings, particularly during the Southwest monsoon period. A high amount of thermal mass without hygroscopic characteristics may have some adverse impacts on the indoor thermal environment, as there is less opportunity to purge the heat stored in it, due to a high minimum temperature, small diurnal temperature range, and predominant cloudy conditions at night. Due to cloudy sky conditions, diffuse radiation is comparatively high, which requires glare control on the openings.

### 4.4. A Framework to Design in Response to a Hot–Humid Climate: Colombo

Table 1 outlines four broad design strategies for the Colombo climate based on the climate analysis performed as climate controls in response to outdoor environmental conditions: temperature, solar radiation, wind, and rainfall. These strategies are targeted to address specific issues related to (dis)comfort.

## 5. Case Study Investigation

This section demonstrates how climatic design strategies, as outlined in Table 1, have been applied to address site-specific climatic challenges and respond to the local ecology. Four projects designed by one of the authors were selected, which are the Eco House (EH), the Green Screen House (GS), the Rock Bungalow (RB), and the Eco Apartments (EA). The case study buildings are a convenient sample based on their exemplary features, availability of information, and access to the site. The data collection method includes designers’ approach and interpretation, observation, and spatial analysis of the buildings and documenting the physical attributes of the design.

### 5.1. Eco House (EH)

The Eco House was completed in 2011. It has a floor area of 260 m^2^ and occupies a 658 m^2^ rectangular plot (Table 2). The design concept, grounded in the principle of “doing more with less,” draws inspiration from the characteristics of a tropical rainforest ecosystem [49]. The direct approach mimics the form, functional integration, and environmental adaptability of native plant leaves, which are viewed as organisms that have evolved optimally for lowland rainforest ecosystems. It draws parallels between plant morphology and building design, particularly in how plants adapt to hot, humid climates. The indirect approach incorporates key design propositions from biomimicry [22], such as multifunctionality, diversity, minimal building footprint, and spatial integration. The three-level house balances light and heavy structural elements and accommodates three bedrooms plus staff quarters. Despite meeting extensive programmatic needs, it retains a minimal footprint, reinforcing ecological integration and sustainable living.

### 5.2. Green Screen House (GS)

The Green Screen House was completed in 2016. It occupies a floor area of 270 m^2^ on a 950 m^2^ lot (Table 2). This house is designed according to the “living with nature” concept, inspired by the canopy structure of a tropical rainforest ecosystem, which addresses the need of rain protection [50].

The Green Screen House, designed as a contemporary residence for a young couple, integrates bedrooms with outdoor recreational spaces, and utilises an open-plan layout around a central void and courtyard. Green-screen walls and full-height doors enhance natural ventilation and minimise glare. Balancing light and heavy architectural forms, the house achieves both spatial dynamism and tranquility while maintaining ecological sensitivity and modern living comfort.

### 5.3. Rock Bungalow (RB)

The Rock Bungalow was completed in 2017. It has a floor area of 288 m^2^ and sits on 2.5 hectares of an undulating site with natural rock boulders and lowland tropical vegetation (Table 2). The design connects two prominent rocks available on the site and maintains local biodiversity, landscaping to create a wild, jungle-like atmosphere [51].

The building follows the natural contours of the site with split-level designs that embrace and showcase the rocky terrain. The overarching concept of “framing and regeneration” guided the integration of the built environment with nature. The architectural form balances lightweight and heavy elements to complement the natural setting.

### 5.4. Eco Apartment (EA)

The Eco Apartments project was completed in 2024. This building responds to the urban typology in the hot–humid climate of Colombo by utilising the concept of “scattered blocks”. It occupies a floor area of 1000 m^2^ on a 696 m^2^ flat land (Table 2). This design mimics characteristics of the lowland tropical ecosystem, particularly how canopy layers facilitate the natural movement of air, wind, rain, and light, regulating heat, temperature, humidity, and glare through passive means.

The medium-density residential development offers four two-bedroom apartments, four single-bedroom apartments, and a shared rooftop entertainment area. Four tower blocks are arranged in a scattered layout, which are interconnected both vertically and horizontally via a central staircase.

### 5.5. Observation and Spatial Analysis of Case Studies

Data collection employed multiple observational techniques to gather qualitative information on the physical and functional characteristics of each building, including layout, spatial configuration, envelope, form, structure, materials, and finishes (Table 2). These methods provided insights into architectural design responding to the local climate while ensuring ecological response in the design solutions. The research method adopts techniques such as direct observation, photographic documentation, behavioural and spatial mapping, field notes, spatial analysis, and contextual observation. To ensure consistency, a structured checklist was developed and applied across all four projects.

### 5.6. Designer’s Approach and Interpretation of the Application of Biomimicry and Biophilic Patterns in the Case Studies

The designer’s approach to the conceptual formulation of the case studies was grounded in the principles of ‘designing with nature’ and ‘doing more with less’, drawing upon the theoretical foundations of biomimicry and biophilia. The framework (Table A1 in Appendix A) incorporated components of the Biomimicry theoretical model [40] together with biophilic design elements [20,42] and biophilic patterns [41]. These informed the integration of natural forms, geometries, colours, textures, vegetation, and visual connections with nature into the built environment. Furthermore, natural elements were strategically aligned with climatic variables, air (ventilation), light (daylighting), and rain (acoustic and thermal regulation) to evoke biophilic experiences of prospect and refuge, both directly and indirectly.

The analytical process involved mapping the ecological characteristics of natural ecosystems to identify strategies for adaptation and integration. These strategies informed the synthesis of architectural form, spatial configuration, and material fabric, thereby enhancing environmental fit [22]. This approach was underpinned by a deeper understanding of ecological integration across ecosystems [52] and was applied across both micro- and macro-contextual scales, including climatic conditions, solar orientation, wind flows, monsoonal rainfall patterns, vegetation cover, and site topography.

In addition, the research engaged with indirect biomimicry approaches, incorporating biomimicry principles [17], ecosystem-level frameworks [52], and biomimicry design propositions [22] as complementary strategies. These were employed in conjunction with biophilic design patterns to guide the formulation of context-responsive and ecologically integrated design outcomes.

**Figure 9:**
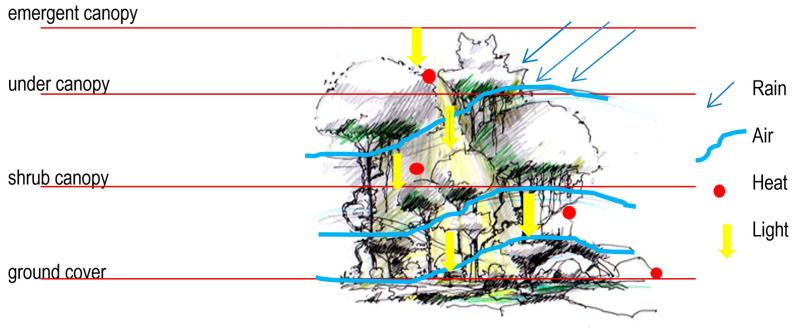
Mimicking a canopy structure in a lowland rainforest [22].

The canopy structure of a lowland rainforest ecosystem (Figure 9), and specifically its constituent organisms, was regarded as an ideal model for interpreting microclimatic conditions across the selected locations [22]. Among these, plant organisms, particularly the morphology of leaves (including their type, size, shape, texture, and patterns), were prioritised due to their static nature, which allows for the study and emulation of adaptive and functional processes in response to environmental stimuli. These morphological and physiological qualities inspired the generation and shaping of architectural forms that respond sensitively to microclimatic contexts [49].

Furthermore, the biophilic patterns inherent in the rainforest canopy were analysed with respect to air movement, temperature regulation, wind dynamics, and light penetration [49,50,51]. This analysis informed the translation of climatic insights (Table A2 in Appendix A) into tangible architectural attributes, including building layout, spatial configuration, envelope design, structural systems, form development, material selection, and surface finishes [18,22,40].

## 6. Bioinspired Climate Control Measures Adopted in the Case Study Buildings

The four case studies were evaluated through designers’ interpretation, spatial analysis, and observation techniques, focusing on climate control measures, ecological integration, and behavioural characteristics. The analysis was guided by the biomimicry theoretical model [40], biophilic patterns [41], and biomimicry design propositions [22]. Insights from Part 1: Climate Design Response informed the identification of four broad strategies (Table 1): i.e., temperature control (TC), humidity control (HC), glare control (GC), and rain protection (RP). These strategies were further analysed for their ecological contributions and behavioural implications, providing a structured basis for understanding the application of bioinspired principles in practice.

### 6.1. Strategies for Temperature Control (TC)

In Colombo, a north–elongated building with a single banked design is preferred. Long eaves projections are favoured for sun control. In addition, existing trees and vegetation can be used as shading elements. The idea is to avoid any additional heat gain through building fabric, which can cause further discomfort for the occupants. If the site topography allows, buildings can be arranged in groups to maximise cooling breezes, while self-shading each other to reduce the thermal load in a hot–humid climatic environment.

**Figure 10:**
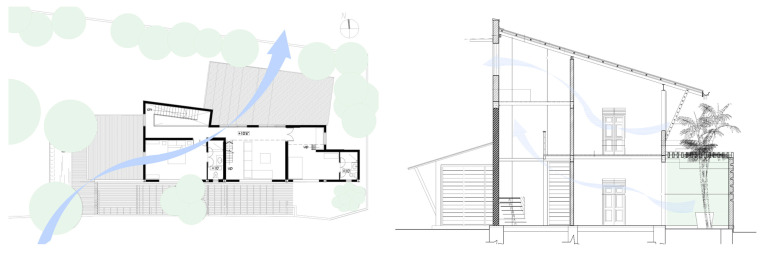
The Eco House upper floor plan and section.

The Eco House (EH) in Colombo (Figure 10) exhibits an ideal orientation. The site allowed the building to elongate on the east-west axis with protruding rectilinear forms, integrated with courtyards on the southern side. The shading strategy was adopted in the early stages by using existing trees on the western side. A well-insulated raked roof and long eaves helped to reduce heat gain from the roof and walls. This house maximised cross-ventilation by having openings at different levels, which are regulated using bamboo blinds to increase wind speed as required. The timber stairs with a void in the center act as a vertical stack and contribute towards the air flow indoors through the stack effect.

**Figure 11:**
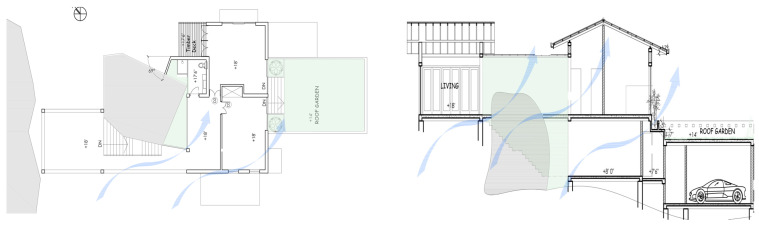
The Rock Bungalow upper floor plan and section.

While the site of the Eco House provided an ideal orientation, the Rock Bungalow (Figure 11) presented many challenges, including unfavourable site orientation due to the site topography, the client’s astronomical requirements, and rocky soil conditions. To achieve the best outcome based on the above-mentioned constraints, the buildingwas oriented to the NE–SW direction, which was not the ideal solution in terms of bioclimatic design (Figure 10). However, allocating the spaces appropriately according to the activities, the bedrooms facing south, and the living space north between two natural boulders with extended verandas helped to reduce the heat gain. This project demonstrates working around the existing constraints and utilising the existing natural features (i.e., boulders and trees) in the building form to create a semi-enclosed courtyard for both natural light and ventilation. Permeable exterior surfaces and water features are used to further cool the landscape. In addition, features such as a veranda and vertical shading devices on the eastern and western sides were incorporated to avoid unwanted heat in living spaces (Figure 10, section). Existing vegetation and the roof garden helped to further reduce heat gain into the bedrooms.

### 6.2. Strategies for Humidity Control (HC) 

In a hot–humid climate, a scattered layout of buildings is preferred to enhance cross-ventilation. However, smaller lots in urban settings may not provide such an opportunity. In such cases, buildings with protruding forms of rectilinear shapes with semi-enclosed courtyards can be beneficial (Figure 9 and Figure 10). Based on the site conditions, the building layout can be changed to manipulate existing vegetation and topography. If the site topography allows, buildings can be arranged in groups to maximise cooling breezes, while shading each other to reduce the thermal load.

The Eco House (Figure 10, ground floor plan) exhibits some interesting spatial configurations. It uses well-shaded protruding forms to maximise the surface area to enhance breeze penetration. The attic space and the stairway support stack ventilation to facilitate indoor air movement (Figure 10, Section).

Spatial configuration plays an important role in mitigating the impact of humidity. It is important to understand the functions of each space when arranging rooms and peripheral activities in a building. Well-shaded northern and southern orientations were used in the houses to accommodate important activity areas such as living, dining and bedrooms. However, indoor moisture-generating activities need to be controlled to avoid an increase in moisture indoors; therefore, the kitchen and bathrooms were separated from the other living areas, and exhaust fans were used to extract humid air outside.

Similarly, the positioning and sizing of the courtyards can be helpful to increase the surface area when the lot does not allow elongation of the building along the east–west axis. The courtyards can be either closed or open based on the intended activities around them. Due to prevailing high-humidity conditions, hygroscopic or breathable building materials such as wood, sun-dried bricks, compressed earth blocks, and cement lime plaster are helpful to regulate moisture indoors. However, contemporary buildings often use cement rendering and apply impervious paints, which restrict moisture absorption by the wall surfaces.

**Figure 12:**
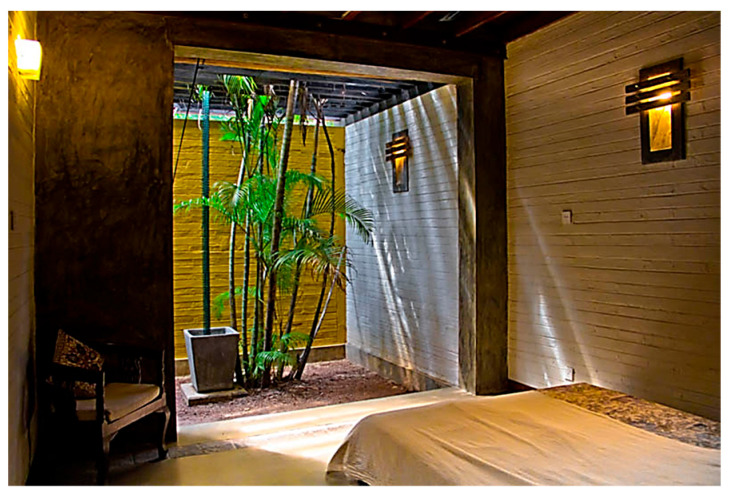
Unplastered earth bricks in the Eco House.

**Figure 13:**
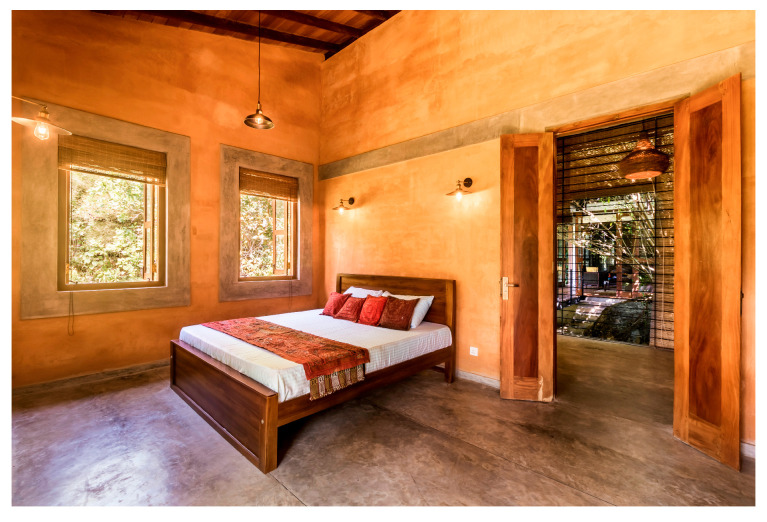
Rammed earth walls absorb moisture in the Rock Bungalow.

**Figure 14:**
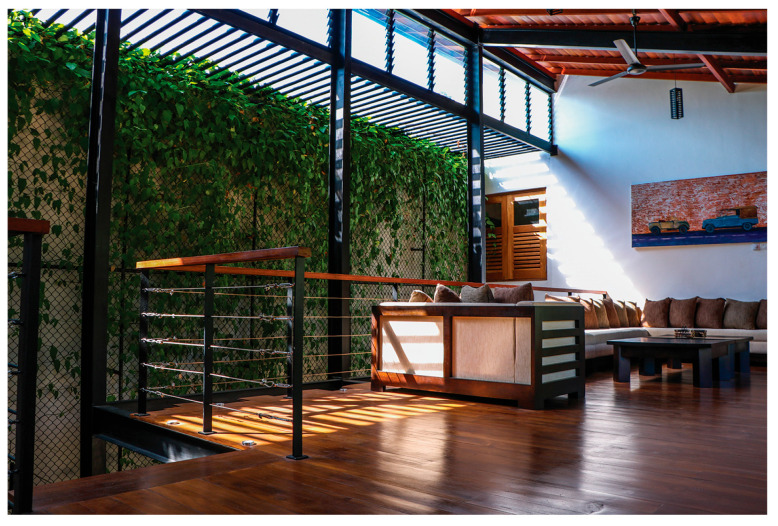
Green screen regulates temperature and humidity in the Green Screen House.

Alternative approaches were adopted in the Eco House, the Rock Bungalow, and the Green Screen House, which included using plaster with fillers, water-based paints, natural finishes such as unplastered earth bricks (Figure 12), and rammed earth walls (Figure 13). Similarly, timber furniture with fabric upholstery, timber ceilings, and cement-rendered flooring was used to regulate indoor moisture levels. Light-coloured and reflective outer surfaces, green screens (Figure 14), moisture-absorbing interior surfaces, and non-toxic water-based paints on interior walls were used where feasible.

### 6.3. Strategies for Glare Control (GC)

Due to diffused and overcast sky conditions in hot–humid climates, it is necessary to control glare to offer visual comfort to the occupants. In the Eco house, railway sleepers were used in the courtyards to cut off direct visual contact with the overcast sky (Figure 12). Vegetation in the courtyards further moderates the glaring impact. The use of bamboo tats in windows helps to minimise direct visual connection to the outside and further contributes to creating a visually comfortable indoors.

**Figure 15:**
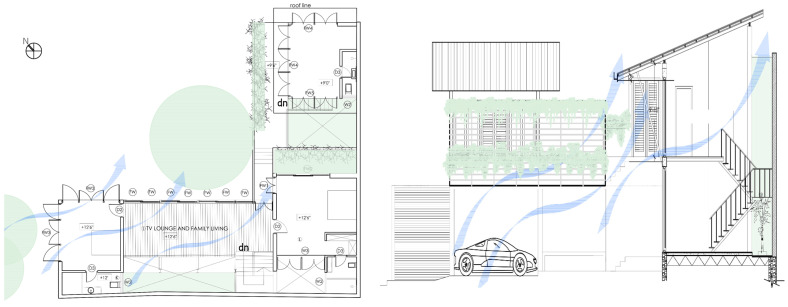
The Green Screen House upper floor plan, and section.

The Green Screen house (Figure 15) utilises various design strategies to enhance the quality of the building envelope. It is an ”L”-shaped building with the main living areas to the north and south and the less frequently used spaces such as the gym and entertainment room located at the western side of the house. This house utilises modifiers such as green screens externally and internally to reduce glare and heat storage in walls. Indirect daylighting was taken into spaces by using vertical screens against the wall or courtyard openings. Natural materials such as coconut ekel blinds, bamboo blinds, and timber and glass louvres and internal and external green screens were used for glare control in walls and pergolas in all projects.

### 6.4. Strategies for Rain Protection (RP) 

Colombo experiences two distinct seasons, rainy and dry, with only slight thermal variations annually. Heavy rainfall and wind movement can be dominating factors to consider in developing the spatial layout. If rooms are to the west, transitional spaces such as balconies, the veranda, and corridors need to be introduced to protect the habitable spaces from the southwesterly rains. The Rock Bungalow (Figure 11) was raised on stilts to allow rainwater runoff through bioswales below the building.

High plinths and raised platforms on stilts were used to avoid water penetration in the building during torrential rain in the monsoon period. Furthermore, large roof overhangs protected rammed walls and windows from heavy rainfall in monsoon seasons.

## 7. Beyond Climate Controls: Adopting Ecological Features and Occupant Behaviour

Climate control measures, along with the ”Strategies for Ecological Integration (EI)”, were employed to establish relationships between climate, ecology, and broader sustainability objectives. Additionally, “Strategies for Occupant Behaviour (OB)” were implemented as low-carbon alternatives to achieve indoor thermal comfort and maintain healthy indoor environmental conditions. These behavioural strategies supported ecological integration by influencing occupant habits to enhance the overall effectiveness of the design.

### 7.1. Strategies for Ecological Integration (EI)

Climate analysis helped to highlight the impact of temperature, humidity, wind movement, glare, and rainfall in building design, whilst ecological integration could further help enhance interactions between these attributes to achieve improved indoor comfort and reduction in embodied and operational carbon emissions.

**Figure 16:**
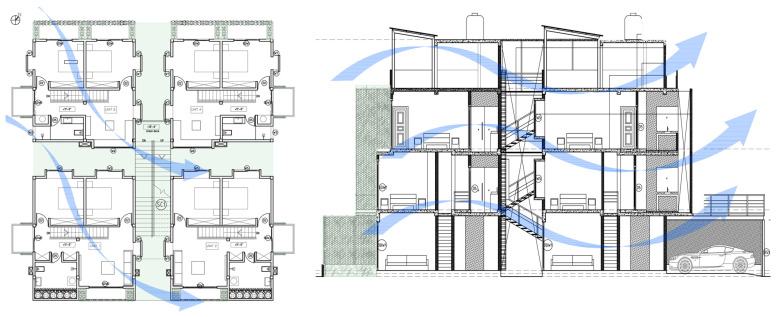
Eco Apartments upper floor plan and section.

In the Eco Apartments (Figure 16), four individual units are separated by an alleyway that runs from NE to SW to facilitate cross-ventilation while self-shading the walls. Green screens avoid unwanted solar heat gain through the walls, and solar panels shade the roof and avoid heat gain through it. The protruding balconies shade windows/walls and deflect wind into the interior spaces. Stairwells further act as ventilation stacks. Protruding balconies and bay windows and green screens further protect and shade the building envelope from rain, glare, and heat gain.

In the Eco Apartments, rainwater is used for the non-potable water demands of the building, as well as for irrigating the plants. The photovoltaic panels are designed as a part of the roofing system to create a parasol roof.

**Figure 17:**
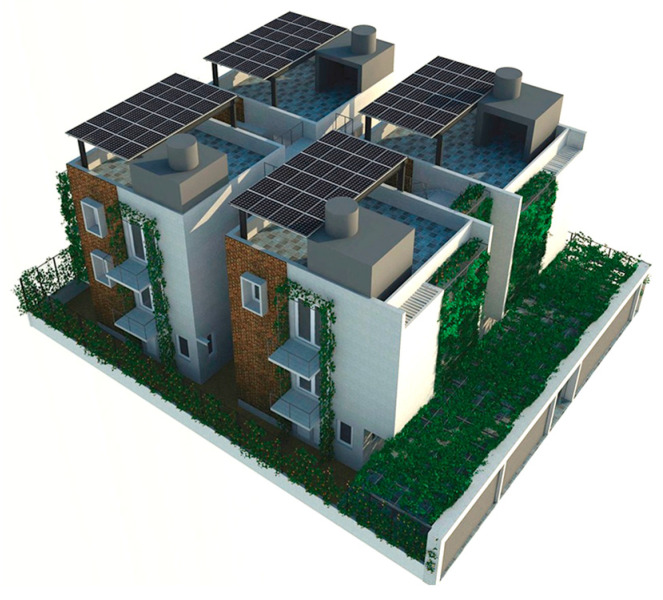
Green screen in the Eco Apartment.

**Figure 18:**
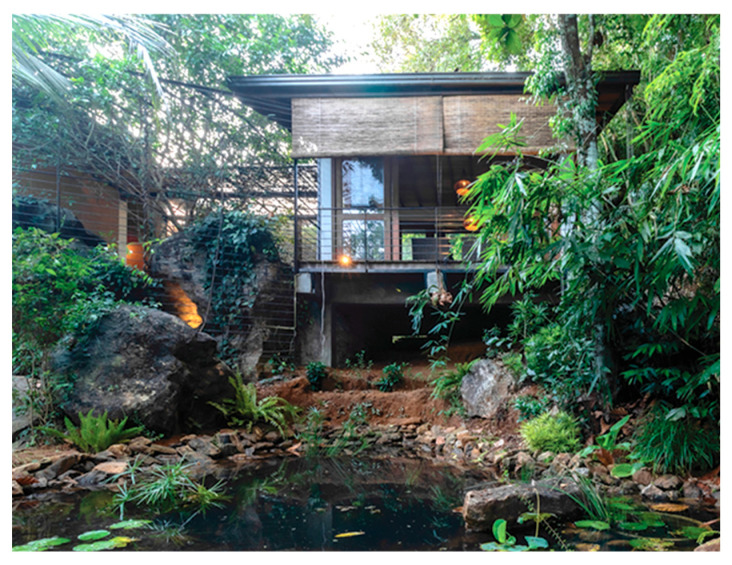
Rain gardens in the Rock Bungalow.

**Figure 19:**
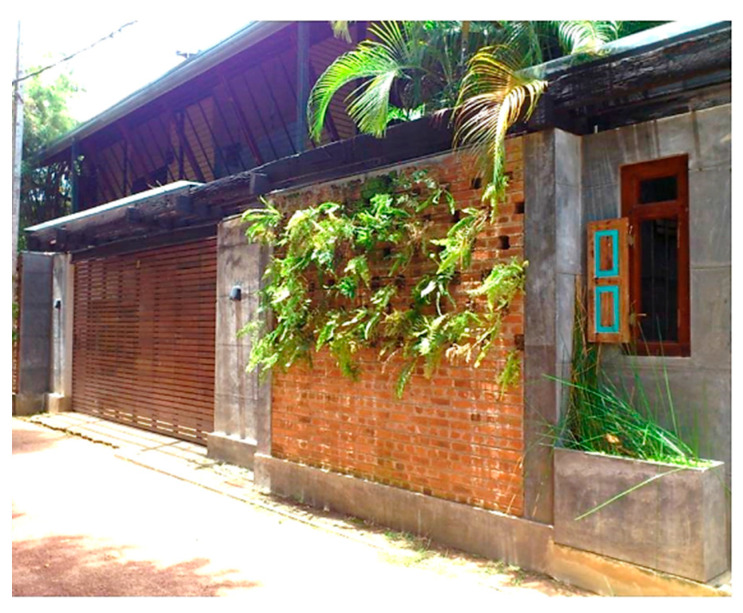
Green wall in the Eco house.

Eco-technological integration could further enhance the environmental quality of bioclimatic design [40]. For example, eco-technologies such as rainwater harvesting tanks, green screens (Figure 17), rain gardens (Figure 18), green walls (Figure 19), photovoltaic panels, grey water systems, septic tanks, and soakage pits act as design modifiers and contribute to enhance the environmental quality of the site and surroundings.

The use of vegetation, mainly shade-providing trees, around the building could improve thermal comfort. Vegetation in courtyards or green screens helps in maintaining indoor air quality. Some plants also act as air purifiers. Research conducted by the National Aeronautics and Space Administration (NASA) found that plants such as bamboo, palm, mother-in-law’s tongue, and peace lily are particularly effective at removing toxic chemicals such as formaldehyde, benzene, and carbon monoxide [53]. Palm trees have been widely used in hot–humid tropics, and they regulate humidity by absorbing moisture through their leaves. Most of the above-mentioned plants are used as modifiers to achieve the expected indoor environmental quality in all the case studies.

### 7.2. Strategies for Occupant Behaviour (OB)

A true BCD requires the active participation of the occupants. A reduction in energy demand in buildings can only be achieved when every occupant embraces a set of environmental values, which enables them to adjust their actions based on changing environmental conditions. For example, in hot–humid environmental conditions, the operation of shading devices/blinds and windows is crucial to maintain acceptable indoor environmental conditions. Opening external shading devices (i.e., tats/louvres) in the evening through to morning and closing them during the daytime in Colombo is beneficial. Similarly, rather than turning on energy-intensive cooling systems (e.g., air conditioners), the occupants should try some simple behavioural adjustments, such as looking for cool spots in the house and using low-energy cooling systems such as fans. These are examples of desirable occupant behaviour in reducing energy consumption. The case study buildings have integrated climate modifying features (i.e., adjustable shading devices, ceiling fans) which can be easily manipulated by the occupants to achieve thermal comfort.

A BCD should not only deliver indoor comfort but also a healthy indoor environment and an ideal fit for its outdoor environment. The occupants should avoid using internal paints, finishes, and furniture which contain toxic gases such as volatile organic compounds (VOCs). Additionally, occupants may use indoor plants to purify the air to maintain a healthy indoor environment.

## 8. Findings

**Figure 20:**
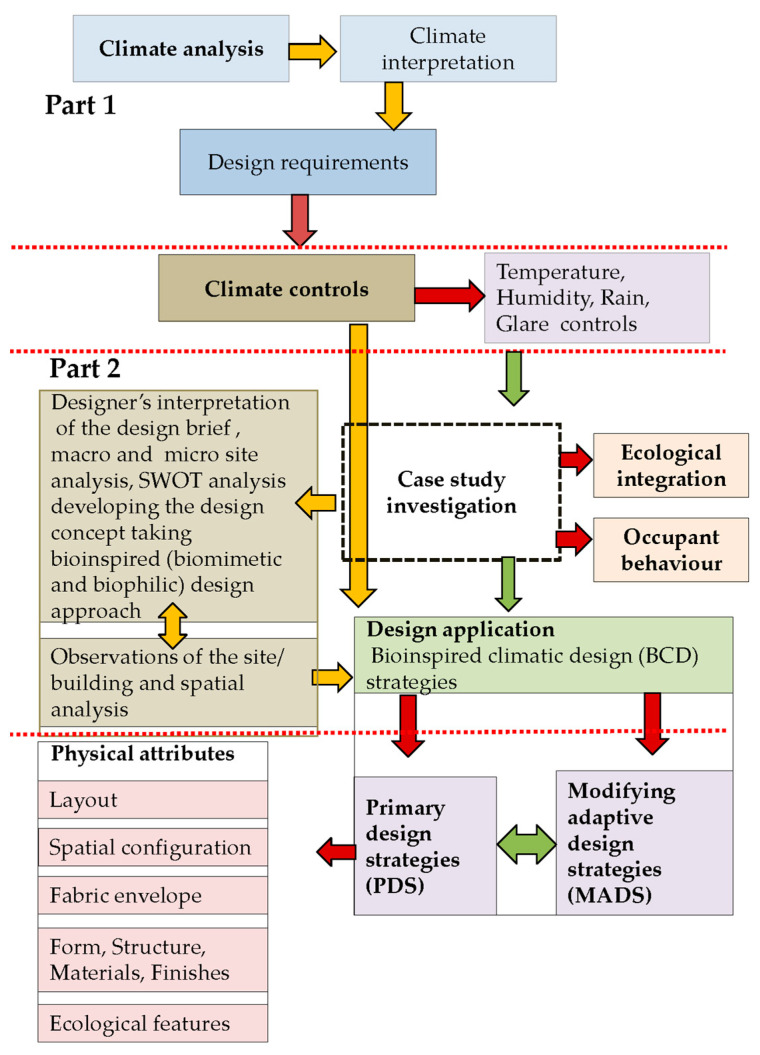
Research findings in response to the methodology.

The evaluation of the case study projects with respect to climate controls, ecological integration, and occupant behaviour strategies (Figure 20) highlighted that it was not always possible to adopt all the design strategies suggested by climate analysis in every project due to the site constraints or the client’s requirements. An ideal site and context may allow designers to adopt most climate-responsive design strategies [54], which can be referred to as ”primary” strategies.

### 8.1. Primary Design Strategies (PDS)

The primary design strategies (PDS) are often derived directly from climate analysis, which is referred to in most of the literature. For Colombo, the PDS are illustrated in Table 2. They are specifically related to the physical attributes of a building, such as the layout, spatial configuration, fabric/envelope, form, structure, and building materials. For example, scattered patterns of buildings, buildings oriented to north and south, and well-shaded windows can be applied in a favourable site. If, for some reason, most primary design strategies cannot be incorporated, the designer can use modifiers to accomplish the goals of bioclimatic design.

### 8.2. Modifying Adaptive Design Strategies (MADS)

Case studies demonstrated that modifying adaptive design strategies (MADS) can often enhance or complement primary strategies by integrating eco-technologies or services and augmenting occupant behaviour. They revealed that these design strategies are also helpful in controlling designs in challenging contexts where primary strategies are impractical and cannot be accommodated due to practical reasons. In addition, it is revealed that ecological integration and occupant behaviour further enhance indoor comfort, i.e., thermal, acoustic, and visual comfort, in most buildings. Some examples of MADS include the use of protruding forms (e.g., balconies) for shading and enhancing breeze penetration, the use of courtyard size and positioning to respond to local climate, the use of split-level to promote air movement around the building, and the use of vegetation or green screens as a shading element on western and eastern facades.

Table A2 in Appendix A provides a summary of the BCD strategies applied in each case study building. The buildings represent a mix of both ideal and challenging site conditions. Through climate analysis, architectural BCD thinking, and spatial observation, the study highlights how design strategies were integrated and combined differently across projects. This comparison helped reveal how each building responded to its specific microclimate and managed climate control through the application of PDS and MADS.

## 9. Proposed Applied BCD Matrix for a Hot–Humid Climate

The observation and spatial analysis of the four case studies demonstrate that with careful manipulation and combinations of primary and modifying adaptive strategies, it is possible to achieve a thermally comfortable ecological design in the hot, humid climate of Colombo. Table 3 illustrates applied BCD strategies for the hot–humid climate of Colombo and identifies relevant climate controls: temperature controls (TC), humidity controls (HC), glare controls (GC), rain projection (RP), strategies of ecological integration (EI), and occupant behaviour (OB). This table uses architectural physical attributes, i.e., layout, spatial configuration, building fabric/envelope, building form, structure, materials, and ecological features to outline applied bioclimatic strategies—primary (P) or modifying adaptive (M)—that correspond to climate controls, ecological integration, and occupant behaviour in response to evaluation of the four case studies (Table A2 in Appendix A).

Findings (Table 3) reveal that PDS are mainly applied in layout planning, spatial configuration, and building envelope/fabric design features, while MADS are primarily used in ecological features, form, materials, structure, and finishes. Most PDS focus on climate controls, whereas ecological integration and behavioural design features fall predominantly under MADS. All ecological features align with ecological integration and fall within the umbrella of MADS.

Analysis in Table A2 in Appendix A shows that all case studies demonstrated a balanced mix of both PDS and MADS in layout design. However, PDS dominated spatial configurations and envelope/fabric design in EH, GH, and RB, with only one strategy applied in EA. Across all four case studies, MADS were more prevalent in ecological features, form, materials, structure, and finishes, with EA exhibiting the highest number of MADS applications compared to PDS.

## 10. Discussions

This study advances climate-responsive architecture in hot–humid contexts through the concept of Bioinspired Climatic Design (BCD), which integrates bioclimatic strategies with principles of biomimicry and biophilia.

### 10.1. Climatic Context and Theoretical Framework

Part 1 identified four monsoon-based seasons in Colombo, each associated with distinct environmental challenges: thermal discomfort, high humidity, glare, and heavy rainfall. From these, four broad climate control strategies were proposed: temperature control (TC), humidity control (HC), glare control (GC), and rainfall protection (RP)

This framework provided a structured basis for evaluating case studies and guiding design responses tailored to local climatic conditions.

### 10.2. Translation into Practice

Part 2 applied this theoretical framework (Table 1) to examine three key domains such as climate controls (TC, HC, GC, RP), Ecological integration (EC), and occupant behaviour (OB), by investigating four residential projects in Colombo using observation and spatial analysis (Table 2), which developed a BCD matrix (Table 3).

Findings show that design strategies were not uniformly applied but were instead adapted to site constraints, client requirements, and ecological opportunities. Ecological features consistently functioned as Modifying Adaptive Design Strategies (MADS), while other attributes—such as form, spatial organisation, and material selection were applied as either Primary Design Strategies (PDS) or MADS depending on project context.

### 10.3. Bioinspired Climatic Design in Practice

The case studies reveal that BCD strategies in hot–humid climates aim to prevent heat gain, regulate indoor moisture, enhance natural ventilation, and mitigate monsoonal rain. These strategies often drew from ecological analogies. For example, the layering of rainforest canopies informed protective building envelopes; courtyards and vertical voids promoted stack ventilation; lightweight breathable fabrics and hygroscopic materials managed humidity; and vegetation and shading reduced glare and solar heat gain.

### 10.4. Flexibility and Contextual Adaptation

A key insight is that successful BCD cannot rely on prescriptive “recipes.” Instead, designers must creatively combine PDS and MADS in response to site-specific conditions. For instance, the Rock Bungalow and Eco Apartments adopted fewer primary strategies due to limited site areas and client constraints, but compensated with adaptive features such as enhanced shading, ventilation, and biodiversity integration. This highlights the importance of flexibility and innovation, where context, ecology, and user behaviour collectively shape design outcomes.

It is important to note that the strategies outlined in Table 3 serve as a reference guide only. Given the variability of site conditions, client expectations, and regulatory frameworks, it is not feasible to apply all strategies within a single building. Instead, BCD must be site-specific, responding to the local microclimate, environmental characteristics, and adaptive behaviour of occupants.

### 10.5. Beyond Climatic Adaptation: Ecological and Biophilic Dimensions

Beyond environmental performance, BCD integrates ecological intelligence into architecture. While biomimicry informed the emulation of natural processes and forms, biophilia guided spatial and material choices that foster human–nature connections.

Climatic variables such as air, light, and rain were employed not only for control but also to enhance sensory and psychological well-being. This aligns with biophilic experiences of prospect, refuge, and immersion in natural processes.

### 10.6. Reinterpreting Rather than Replicating

The findings underscore that BCD is not about replicating vernacular forms but about reinterpreting natural strategies to address contemporary needs for comfort, density, and regulatory compliance. By embedding ecological and biophilic principles into climate-responsive strategies, BCD offers a flexible, scalable, and low-carbon framework for architecture in hot–humid climates.

## 11. Conclusions

This study positions Bioinspired Climatic Design (BCD) as a robust framework for climate-resilient and ecologically integrated architecture in hot–humid regions. By drawing on bioclimatic principles alongside biomimicry and biophilia, BCD enables passive and adaptive strategies that reduce carbon emissions while enhancing comfort and ecological performance.

Findings demonstrate that while passive design principles provide a foundation, successful application depends on the creative combination of Primary Design Strategies (PDS) and Modifying Adaptive Design Strategies (MADS) tailored to site-specific challenges. Case studies show that MADS are critical for addressing adverse climatic impacts without reliance on energy-intensive systems, while also integrating ecological features such as biodiversity, stormwater management, resource conservation, and low-carbon materials.

The research underscores the need for location-specific BCD frameworks that align climate control, ecological integration, and occupant comfort. The methodological approach presented here is transferable to other climatic contexts, supporting the development of tailored, ecologically grounded design strategies which have the potential to be incorporated into building codes or standards in hot–humid regions.

By situating BCD at the intersection of vernacular knowledge, ecological intelligence, and contemporary climate-responsive design, this study contributes to sustainable architecture discourse. BCD emerges as a forward-looking pathway for low-carbon, resource-efficient, and resilient architecture, offering scalable solutions for hot–humid regions and beyond.

## Figures and Tables

**Table 1:** Proposed framework for climatic design response for Colombo.

Outdoor Environmental Conditions	(Dis)comfort Conditions	Broad Design Strategies
**Thermal environmental conditions** Humid (72% of the time/year). Hot–humid (28% of the time/year).	High humidity is responsible for severe to extremely severe levels of heat discomfort.	Strategies for temperature control Strategies for humidity control Strategies for glare control Strategies for rain protection
**Solar radiation/sky conditions** High level of diffuse radiation. Predominantly overcast sky condition.	Additional heat gains due to direct exposure to solar radiation further exacerbate discomfort. Visual discomfort due to overcast sky.	
**Wind** Wind speed picks up in the afternoon. Low wind speeds in the evening through to morning.	Wind movement helps to achieve cooling sensation and contributes to perceived thermal comfort.	
**Rainfall** Mostly rainy and daily rainfall exceeds 100 mm occasionally.	Humidity level increases significantly. Heavy rain can cause waterlogging and localised flooding.	

**Table 2:** Observation and spatial analysis: site context and attributes of the case study buildings.

Physical Attributes/Case Study	Layout	Spatial Configuration	Envelope/Fabric	Form, Structure, Materials, Finishes
**Case Study 1 (CS1):** **EH** Location: 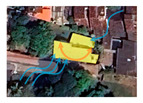 (Google Earth image) Lat: 6°51′20.6″ N Long: 79°53′07.0″ E Land area: 658 m^2^ Building area: 260 m^2^	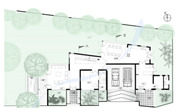	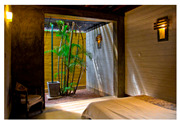	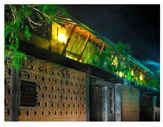	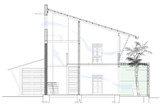 Section
	The site is oriented north-to-south with a staggered perimeter. The land is flat with gravel and hard soil, and trees taller than 3 m are found on the northern and western sides.	The house has a heavy (concrete slab) ground floor and a light (timber) upper floor. Bedrooms are on the south side, while the living and dining areas face north. It has hardwood pergolas made from railway sleepers, and three courtyards on the southern side.	The house has a Zincalum roof with roof insulation using double-sided foil. Steel grilles are placed on the southwest and northeast sides. Bamboo blinds are used for shading, and there is a large overhang on the southwest. Polycarbonate canopies are provided, and the site is enclosed with a green boundary wall.	The house has a protruded form with load-bearing brick walls and steel on the upper floor. A hardwood stair with a void connects the levels. The roof has exposed rafters with a raked timber ceiling. A reused double brick wall is part of the design. The finishes include rough plaster, exposed bricks, and integrated services. Rain chains are used, and the exterior shows clay colour, grey cement render, and white surfaces.
**Case Study 2 (CS2):** **GS** Location: ** 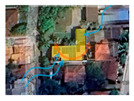 ** (Google Earth image) Lat: 7°05′10.4″ N Long: 79°53′23.1″ E Land area: 950 m^2^ Building area: 270 m^2^	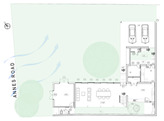	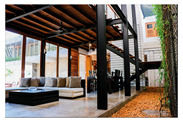	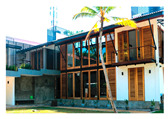	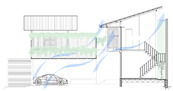 Section
	The site has a northeast–southwest orientation and features an L-shaped perimeter. The topography is flat, and the soil is gravelly and hard. Vegetation includes a mango tree approximately 4 m tall, along with coconut trees that exceed 6 m in height.	The ground floor has heavy mass, and the upper floor is lightweight. Bedrooms and the living and dining areas face north. There are three courtyards on the northeast, southeast, and eastern sides. Hardwood stairs with a void connect the levels.	The house uses double brick masonry walls with hardwood louvre windows and doors. Zincalum cantilevered balconies and a large overhang are provided. Glass louvres are placed on the northeast side. The roof has double-sided foil insulation, and both the interior and exterior feature steel mesh green screens.	The design includes voids and double-height spaces. It has an exposed timber rafter roof with a raked timber ceiling. Services are integrated into the design, and rain chains are provided. The finishes use white colour, with grey cement-rendered floors and exterior white walls.
**Case Study 3 (CS3):** **RB** Location: ** 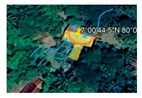 ** (Google Earth image) Lat: 7°00′44.5″ N Long: 80°06′18.3″ E Land area: 2.5 h Building area: 288 m^2^	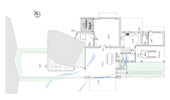	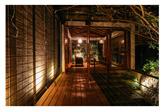	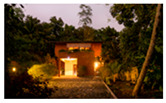	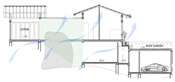 Section
	The site has a northeast–southwest orientation. The house is designed with a scattered perimeter. The topography is hilly and undulating, and the soil is rocky and gravelly. Vegetation is sparse, with no trees on the southwest side, while trees over 4 m are present on the northern side. The western side of the site is rocky.	The ground floor and upper floor have a combination of heavy and lightweight construction. The bedrooms are oriented to the north, while the living area and the dining area faces southwest. Courtyards are located on the northern side, and the living area is situated between two natural rocks.	The house features rammed earth walls with a zinc aluminium roof, which is insulated using double sided foil. Windows and doors are oriented toward the southwest to capture prevailing winds. The roof garden faces southeast, complemented by large polycarbonate overhangs. Steel-framed canopies and pergolas are integrated.	The building is a scattered, split-level design supported on concrete stilts, with rubble-packed retaining walls. Outdoor paving is permeable, and the interior features double-height spaces. The roof has exposed rafters with a raked timber ceiling, while services are integrated throughout. Rain chains are incorporated, and the exterior combines clay-coloured and grey cement-rendered surfaces.
**Case Study 4 (CS4):** **EA** Location: 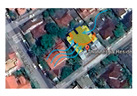 (Google Earth image) Lat: 6°58′55.06″ N Long: 79°55′39.85″ E Land area: 658 m^2^ Building area: 1000 m^2^	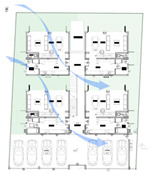	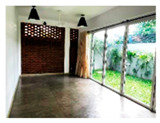	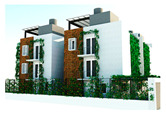	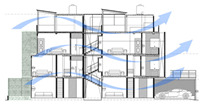 Section
	The site has a northeast–southwest orientation. The apartment uses a scattered perimeter in a flat topography. The site is a tight urban block with no vegetation.	The building combines heavy and lightweight construction. Being an apartment building, habitable spaces are oriented to all possible sides. Stairs are centrally located. Voids and double-height spaces are positioned between the cubic volumes, enhancing openness and connectivity.	The design features glass louvres and an exposed single-brick wall, along with perforated patterned walls and green screens. Cantilevered balconies and bay windows add depth, while steel grilles provide security. The roof incorporates polycarbonate panels with bamboo shading, and portions of the roof slab are covered with photovoltaic panels.	The design consists of four segregated cubes. Outdoor spaces feature permeable gardens and bio-retention fields, with integrated services and rainwater harvesting tanks. Green walls are incorporated on the western and eastern sides.

**Table 3:** Applied BCD matrix for a hot–humid climate.

Applied BCD Strategies/Attributes	Climate Controls, Ecological Integration, and Occupant Behaviour ^1^					
**Layout**	**TC**	**HC**	**GC**	**RP**	**EI**	**OB**
Use scattered patterns of buildings, rather than a single block.	P	P				
Adopt building orientation preferably to north–south.	P					
Embed shading strategy at an early stage of building layout.	P		P			
Use existing trees as wind breakers and shading devices.	P	P		P		
Use permeable soft surfaces and integrate water features in landscaping.	.M				M	
Use protruding forms for shading and enhance breeze penetration.	M	M				
Use courtyard building forms and north–south boundaries.	M	M			M	
**Spatial Configuration**	**TC**	**HC**	**GC**	**RP**	**EI**	**OB**
Adopt single-banked rooms to enhance cross-ventilation.	P	P				
Provide windows on at least two walls to enhance cross-ventilation.		P				
Use split-level planning to promote air movement around the building.		M				
Arrange rooms in different orientations based on the activities performed on those spaces.	P	P				
Segregate moisture-generating activity spaces such as the kitchen and wet areas from main living spaces to address humidity issues.		P				
Provide high ceilings and openings at higher levels to flush out hot air accumulated near the ceiling space.	P					
Locate services and transitional activities on the eastern/western sides.	P					
Use high ceilings with exposed rafters.	P					
**Fabric/Envelope**	**TC**	**HC**	**GC**	**RP**	**EI**	**OB**
Arrange large openings on north and south orientations which can be easily shaded by eaves.	P					
Minimise glazing in the west and shade western windows/openings.	P					
Maximise ventilation using cross-ventilation and stack ventilation.	P					
Use flexible louvres, grilles in windows for glare control. and maximise ventilation indoors.	P	P	P			
Use vegetation or green screens and other building forms of shading elements on western and eastern facades.	M				M	
Introduce pergolas, verandas, balconies, terraces, courtyards, and patios to create semi-outdoor spaces to shade the walls and enhance ventilation.	P	P		P		
Provide wide overhangs to protect walls and windows from sun and heavy rainfall in monsoon seasons.	P		P	P		
**Form, Structure, Materials, and Finishes**	**TC**	**HC**	**GC**	**RP**	**EI**	**OB**
Use hygroscopic building materials (e.g., timber, sun dried bricks, compressed rammed earth, lime plaster) to moderate indoor moisture level.		M				
Use high plinths or raised platforms on stilts to avoid ground radiation and water penetration during torrential rain in the monsoon period.	P			P		
Use a well-insulated pitched roof with long overhangs to reduce heat gain and rain control.	P			P		
Introduce a parasol roof to avoid unnecessary heat gain through an uninsulated flat roof and protect it from rain.	P			P		
Arrange (size and position) courtyard(s) to enhance ventilation.	M	M			M	
Use lightweight but well-insulated external building fabric.	P					
Use photovoltaic panels as shading devices if possible.	M				M	
Use ceiling fans in all living and sleeping spaces to increase air speed when indoor air is stagnant.	M	M				M
Explore energy recovery ventilation (ERV) or heat recovery ventilation (HRV) before selecting air conditioners.	M	M				
Operate flexible elements appropriately, e.g., opening and closing windows/internal shading devices, etc.	M	M	M	M	M	M
Actively seek cool spots or use low-power cooling appliances before using air conditioners.	M	M				M
Use moisture-absorbing indoor furnishings and furniture.		M				M
Use natural cleaning ingredients and avoid toxic chemicals.						M
**Ecological Features**	**TC**	**HC**	**GC**	**RP**	**EI**	**OB**
Place rainwater harvesting tanks to the western and eastern sides.	M				M	
Combine seepage beds and bio-retention areas with rain gardens to allow ground recharge.	M			M	M	
Use solar panels as a roof/canopy for shading.	M		M			
Treat grey water onsite and maintain a closed-loop system if possible.					M	
Use rain chains to downpipes and bioswales (open drains filled with pebbles and perforated pipes).					M	
Use native plants (e.g., bamboo, peace lilies, and reed palm) in the courtyards and green screens to regulate humidity.		M	M		M	
Use indoor plants as air purifiers, medicinal herbs, spice plants, and insect repellents.	M				M	M
Use host plants to attract birds, bees, and butterflies to increase biodiversity.	M				M	
Use locally available natural materials such as coconut ekel, bamboo tats, and timber louvres as indoor screens.	M	M	M	M	M	
Use water-based paints or low/no VOC paints.					M	
Integrate eco technologies and services with physical attributes.	M				M	

^1^ Climate controls, ecological integration, and occupant behaviour attributes are identified as Temperature control (TC), Humidity control (HC), Glare control (GC), Rainfall protection (RP), Ecological integration (EI), Occupant behaviour (OB), MADS (M), and PDS (P).

## Data Availability

The raw data supporting the conclusions of this article will be made available by the authors on request.

## Abbreviations

The following abbreviations are used in this manuscript:

BCD	Bioinspired Climatic Design
TC	Temperature Control
HC	Humidity Control
GC	Glare Control
RP	Rain Protection
EI	Ecological Integration
OB	Occupant Behaviour
PDS	Primary Design Strategies
MADS	Modifying Adaptive Design Strategies
EH	Eco House
GS	Green Screen House
RB	Rock Bungalow
EA	Eco Apartment

## Appendix A

**Table A1:** Biomimicry theoretical framework [22].

Scale of Application	Design Process	Direct Approach: Specific Mimicking	Indirect Approach: General Mimicking
**Ecosystem** How does it fit with the whole?	**Categorization** What is the type of classification?	Type of species, physical characteristics, climatic zones, relationship between species, size & form variations	Identification of building types, types of users, size variations, form variations, relationship with users &organisms, climatic zones
**Process** How does it perform and how is it made?	**Functional Integration** What are the innovative strategies?	Hierarchy of functions: primary, secondary, techniques physical characteristics, mechanisms, patterns, behaviour patterns, needs, communication, organisation	Users & user needs, hierarchy of functions: primary, secondary functions, techniques, physical characteristics, mechanisms, user behaviour, patterns, needs, occupancy, communication
	**Environmental Adaptation** What are the innovative strategies?	Macro & micro- environment, physical characteristics, habitat, topography, macro & micro-climate: wind, sun path, temperature, humidity, rainfall	Macro & micro-environment, physical characteristics, habitat topography, macro & micro-climate: wind, sun path, temperature, humidity, rainfall
**Form** What is the shape?	**Innovation of form** What is the expression?	Design fundamentals: lines, shape, texture, colour, patterns, geometric progression: module, unit to whole, scale and proportions	Design fundamentals: lines, shape, texture, colour, patterns, geometric progression: module, unit to whole, scale and proportions

**Table A2:** Applied BCD matrix in case studies for the hot–humid climate.

Applied BCD Strategies/Attributes	Climate Controls, Ecological Integration, and Occupant Behaviour ^1^						CS1	CS2	CS3	CS4
**Layout**	**TC**	**HC**	**GC**	**RP**	**EI**	**OB**	**EH**	**GS**	**RB**	**EA**
Use scattered patterns of buildings, rather than a single block.	P	P					X		X	X
Adopt building orientation preferably to north–south.	P						X			
Embed shading strategy at an early stage of building layout.	P		P				X	X	X	X
Use existing trees as wind breakers and shading devices.	P	P		P			X		X	
Use permeable soft surfaces and integrate water features in landscaping.	M				M		X	X	X	X
Use protruding forms for shading and enhance breeze penetration.	M	M					X		X	X
Use courtyard building forms, north–south boundaries.	M	M			M		X	X		
**Spatial Configuration**	**TC**	**HC**	**GC**	**RP**	**EI**	**OB**	**EH**	**GS**	**RB**	**EA**
Adopt single-banked rooms to enhance cross-ventilation.	P	P					X	X	X	
Provide windows on at least two walls to enhance cross-ventilation.		P					X	X	X	X
Use split-level planning to promote air movement around the building.		M							X	
Arrange rooms in different orientations based on the activities performed on those spaces.	P	P					X	X	X	
Segregate moisture-generating activity spaces such as the kitchen and wet areas from main living spaces to address humidity issues.		P					X	X	X	
Provide high ceilings and openings at higher levels to flush out hot air accumulated near the ceiling space.	P						X	X	X	
Locate services and transitional activities on the eastern/western sides.	P						X	X	X	
Use high ceilings with exposed rafters.	P						X	X	X	X
**Fabric/Envelope**	**TC**	**HC**	**GC**	**RP**	**EI**	**OB**	**EH**	**GS**	**RB**	**EA**
Arrange large openings on north and south orientations which can be easily shaded by eaves.	P						X			
Minimise glazing in the west and shade western windows/openings.	P						X	X		**X**
Maximise ventilation using cross-ventilation and stack ventilation.	P						X	X		
Use flexible louvres, grilles in windows for glare control, and maximise ventilation indoors.	P	P	P				X	X		**X**
Introduce pergolas, verandas, balconies, terraces, courtyards, and patios to create semi-outdoor spaces to shade the walls and enhance ventilation.	P	P		P			X	X	X	**X**
Use vegetation or green screens and other building forms of shading elements on western and eastern facades.	M				M		X	X		
Provide wide overhangs to protect walls and windows from sun and heavy rainfall in monsoon seasons.	P		P	P			X	X		
**Form, Structure, Materials, and Finishes**	**TC**	**HC**	**GC**	**RP**	**EI**	**OB**	**EH**	**GS**	**RB**	**EA**
Use hygroscopic building materials (e.g., timber, sundried bricks, compressed rammed earth walls, lime plaster) to moderate indoor moisture level.		M							X	
Use high plinth or raised platforms on stilts to avoid ground radiation and water penetration during torrential rain in the monsoon period	P			P			X	X	X	X
Use well-insulated pitched roof with long overhangs to reduce heat gain and rain control.	P			P			X	X		
Introduce a parasol roof to avoid unnecessary heat gain through an uninsulated flat roof and protect it from rain.	P			P					X	X
Arrange (size and position) courtyard(s) to enhance ventilation.	M	M			M		X	X		
Use lightweight but well-insulated external building fabric.	P									
Use photovoltaic panels as shading devices if possible.	M				M					X
Use ceiling fans in all living and sleeping spaces to increase air speed when indoor air is stagnant.	M	M				M	X	X	X	X
Explore energy recovery ventilation (ERV) or heat recovery ventilation (HRV) before selecting air conditioners.	M	M								X
Operate flexible elements appropriately, e.g., opening and closing windows/internal shading devices, etc.	M	M	M	M	M	M	X	X	X	X
Actively seek cool spots or use low-power cooling appliances before using air conditioners.	M	M				M				X
Use moisture-absorbing indoor furnishings and furniture.		M				M	X	X	X	X
Use natural cleaning ingredients and avoid toxic chemicals.						M	X	X	X	X
**Ecological Features**	**TC**	**HC**	**GC**	**RP**	**EI**	**OB**	**EH**	**GS**	**RB**	**EA**
Place rainwater harvesting tanks to the western and eastern sides.	M				M				X	X
Combine seepage beds and bio-retention areas with rain gardens to allow ground recharge.	M			M	M		X	X	X	X
Use solar panels as a roof/canopy for shading.	M		M							X
Treat grey water onsite and maintain a closed-loop system if possible.					M					X
Use rain chains to downpipes and bioswales (open drains filled with pebbles and perforated pipes).					M		X	X	X	
Use native plants (e.g., bamboo, peace lilies and reed palm) in the courtyards and green screens to regulate humidity.		M	M		M		X	X	X	X
Use indoor plants as air purifiers, medicinal herbs, spice plants, and insect repellents.	M				M	M	X		X	
Use host plants to attract birds, bees, and butterflies to increase biodiversity.	M				M				X	X
Use locally available natural materials such as coconut ekel, bamboo blinds and timber louvres as indoor screens.	M	M	M	M	M		X	X	X	X
Use water-based paints or low/no VOC paints.					M		X	X	X	X
Integrate eco technologies and services with physical attributes.	M				M					

^1^ Climate controls, ecological integration, and occupant behaviour attributes are identified a, Temperature control (TC), Humidity control (HC), Glare control (GC), Rainfall protection (RP), Ecological integration (EI), Occupant behaviour (OB), MADS (M) and PDS (P).
